# Low-level prenatal lead exposure and infant sensory function

**DOI:** 10.1186/s12940-016-0148-6

**Published:** 2016-06-07

**Authors:** Monica K. Silver, Xiaoqing Li, Yuhe Liu, Ming Li, Xiaoqin Mai, Niko Kaciroti, Paul Kileny, Twila Tardif, John D. Meeker, Betsy Lozoff

**Affiliations:** Department of Environmental Health Sciences, University of Michigan, 1415 Washington Heights, Ann Arbor, MI 48109 USA; Department of Pediatric Ophthalmology, Peking University First Hospital, 8 Xishiku St., Xicheng, Beijing, 100034 China; Department of Otolaryngology, Peking University First Hospital, 8 Xishiku St., Xicheng, Beijing, 100034 China; Department of Pediatrics, Peking University First Hospital, 8 Xishiku St., Xicheng, Beijing, 100034 China; Center for Human Growth and Development, University of Michigan, 300 North Ingalls St., Ann Arbor, MI 48104 USA; Department of Psychology, Renmin University, 59 Zhongguancun St., Haidian, Beijing, 100872 China; Department of Biostatistics, University of Michigan, 1415 Washington Heights, Ann Arbor, MI 48109 USA; Department of Otorhinolaryngology, University of Michigan, 1500 E. Medical Center Dr., Ann Arbor, MI 48109 USA; Department of Psychology, University of Michigan, 530 Church St., Ann Arbor, MI 48109 USA

**Keywords:** Lead, Auditory processing, Visual acuity, Infants, Neonates

## Abstract

**Background:**

Lead is a pervasive neurotoxicant that has been associated with poorer cognitive, behavioral, and motor outcomes in children. The effects of lead on sensory function have not been well characterized. The aim of this study was to assess the effects of prenatal lead exposure on infant sensory function, as measured by auditory brainstem response (ABR) and grating visual acuity (VA).

**Methods:**

Lead was measured in maternal blood in mid- and late-pregnancy (mean gestational age = 15.5 and 39.0 weeks, respectively) and umbilical cord blood in a cohort of full-term infants in rural northeastern China. ABR latencies (peaks I, III, V) were measured in newborns during unsedated sleep (*n* = 315). The ABR central-to-peripheral (C-P) ratio was calculated as the ratio between the III-V and I-III interpeak intervals. VA was measured in 6-week-olds using Teller Acuity Cards (*n* = 1019) and assigned as the narrowest grid the infant fixated on. Multivariate linear regression was used to evaluate relationships between tertiles of mid-pregnancy, late-pregnancy, or cord lead and newborn ABR or 6-week VA.

**Results:**

Higher late-pregnancy lead levels were associated with higher ABR C-P ratios and lower VA. In covariate-adjusted analyses, mean C-P ratios were 4.6 and 3.2 % higher in infants whose mothers had lead > 3.8 μg/dL and lead = 2–3.8 μg/dL, respectively, than for infants whose mothers had lead < 2 μg/dL (*p*-trend =0.002). In adjusted analyses for VA, mean scores were 8.5 and 7.2 % lower for maternal lead > 3.8 μg/dL and lead = 2–3.8 μg/dL, respectively, compared to lead < 2 μg/dL *(*p*-*trend =0.009).

**Conclusion:**

Auditory and visual systems maturation appears delayed in infants with higher prenatal lead exposure during late-pregnancy, even at relatively low levels. Both systems start myelinating in late gestation and mature rapidly in infancy. Higher ABR C-P ratio and lower grating VA scores suggest effects of low-level lead exposure on sensory system myelination.

**Electronic supplementary material:**

The online version of this article (doi:10.1186/s12940-016-0148-6) contains supplementary material, which is available to authorized users.

## Background

Lead is a naturally occurring element that has numerous industrial uses. Despite lead abatement successes in much of the developed world through the phasing-out of leaded paints and gasoline, lead remains a large global public health problem [1]. In the U.S. it can still be found in aviation gas, ammunition, and lead-acid car batteries. Many older houses still have leaded paint and pipes. In developing or transitional countries, such as China, it is still used in decorative paints and glazes, children’s toys, gasoline, cosmetics, and much more [2]. Lead’s widespread use has resulted in environmental contamination and human exposure via multiple pathways. Non-occupational exposure most commonly occurs from ingestion of lead-contaminated dust, water, or food [3].

Pediatric lead exposure has been a major public health concern for decades, because lead is highly and selectively toxic to the central nervous system (CNS). Many neurodevelopmental and neurobehavioral effects of childhood and fetal lead exposure have been documented. Childhood lead exposure has been associated with lower IQ and cognitive test scores, more behavioral abnormalities, decrements of executive and motor functions, and altered memory and language, inattention, aggression, and other neurological deficits [4–6]. The fetus is especially at risk, since lead has the ability to cross the placental and blood brain barriers [3, 4]. Additionally, maternal bone turnover during pregnancy may release lead from bone, thereby further exposing the developing fetus [7]. During the fetal period, the brain is undergoing rapid growth and maturation, making it highly susceptible to potentially long-term effects of lead exposure. Given this rapid development, the timing of exposure is of particular importance [8–10], though many studies are limited to only one measurement, which may not accurately represent exposure at sensitive stages.

Despite a growing body of knowledge about the cognitive, behavioral, and motor effects of pediatric lead exposure, much less is known about how lead may affect sensory functions, such as visual and auditory function. Visual and auditory system development in infancy provides the foundation for many learning processes in infancy and childhood, such as development of communication, language, and reading skills [11, 12]. Prenatal lead exposure may alter the timing of sensory systems myelination in the brain, potentially leading to detrimental long-term effects on learning or other cognitive functions later in childhood. Epidemiological studies have examined childhood lead exposure and auditory brainstem response with inconsistent findings [13–23], and only three looked at prenatal lead exposure [18, 21, 22]. We were unable to identify any human studies that investigated prenatal lead exposure and grating visual acuity.

Therefore, we assessed the impact of prenatal lead exposure at three different time points during pregnancy on infant sensory function, as measured by auditory brainstem response (ABR) and grating visual acuity (VA). These measures provide non-invasive ways of assessing auditory and visual system maturation and, indirectly, myelination, since both systems rapidly myelinate during infancy.

## Methods

### Ethics statement

All study protocols and procedures were approved by the appropriate ethics committees of both the University of Michigan and Peking University First Hospital. Signed, informed consent was obtained from parents following a thorough explanation of all procedures.

### Study sample

Study participants were healthy pregnant women, aged 18 years or older, with a singleton pregnancy and no major complications, who resided in Sanhe County, Hebei Province, China. Women were recruited at their initial prenatal visit at Sanhe City Maternity and Child Health Institute, from November 2009 to November 2011, as part of a study of early iron deficiency and neurodevelopment.

### Lead

Whole blood for lead analysis was collected from the mothers at two time points during pregnancy, at enrollment and at or near term, and in cord blood at birth. Maternal blood (5 mL) was obtained by venipuncture and cord blood samples (5 mL) by sterile needle puncture immediately after cord clamping. Blood was collected in trace-metal free tubes treated with EDTA and stored at −20 C until analysis.

Whole blood samples (40 μL) were analyzed for lead using atomic absorption spectrometry (AAS) using a BH2100S tungsten atomizer atomic absorption spectrometer (Beijing Bohui Innovation Technology Co., Ltd., Beijing, China) in the clinical laboratory of the Pediatrics department of Peking University First Hospital (Beijing, China). Blood samples were observed for signs of clotting prior to analysis and none were detected. Blood samples were measured once, but if lead was determined to be >10 μg/dL, samples were re-run and the average of the two measurements was used. The limit of detection (LOD) of the method was 1.0 μg/dL, which was calculated as 3 times the standard deviation of the blank. The limit of quantification (LOQ) for the method was 2.0 μg/dL. The lab participates in the National System of External Assessment of the Quality of Results, an external quality assurance program conducted by China’s National Center for Clinical Laboratories (NCCL). Internal quality control included running blanks and certified reference materials (Chinese national standard: GBW (E) 09003336 [Beijing Bohui Innovation Technology Co., Ltd., Beijing, China] at concentrations of 5, 10, 30 μg/dL) in parallel with study samples (every 10 samples) for each batch. Quality control analysis yielded coefficients of variation ranging from 8.2 to 12.1 %.

Based on the distributions of the blood lead concentrations, and the relatively high LOQ (2 μg/L), we categorized lead roughly into tertiles for analysis (<2, 2-3.8, >3.8 μg/dL for pregnancy lead time points and <2, 2-3.2, >3.2 μg/dL for cord lead).

### Auditory Brainstem Response (ABR)

ABR, also known as brainstem auditory evoked potentials (BAEPs), measures the brain’s electrical activity following an auditory stimulus by quantifying the progressive activation of different points along the auditory pathway. ABRs in infants consist of three prominent peaks, corresponding to distal cochlear nerve activation (wave I), activation of the cochlear nuclei (wave III), and the nucleus of the lateral lemniscus (wave V) [24, 25]. The rapid decrease in ABR interpeak latencies (and thus, increased speed of transmission) observed during early development in infancy is directly attributable to increasing myelination of the auditory pathways in the infant brain [26, 27]. Less decrease in the interpeak latencies in the central (III-V interval), relative to the more peripheral components (I-III interval), of the auditory pathway is considered to be an indicator of impaired myelination [28].

ABR was measured in a subset of 391 newborns (average two days old) during unsedated sleep using a Biologic Navigator (Bio-Logic Systems Corp., Mundelein, IL)/Traveler evoked potential system. Infants underwent ABR testing with a standard hearing screening protocol at 30 dB and a second forward-masking protocol with varied intervals (8, 16, or 32 ms) between pairs of monophasic clicks at 80 dB.

Stimuli for the hearing screening test were a series of square wave rarefaction clicks with a duration of 100 μs, delivered to each ear by means of insert transducers at a rate of 31.3/sec and intensity of 30 dB, nHL. ABR was recorded by surface silver/silver chloride electrodes attached to infant's foreheads using adhesive tabs (midline below the hairline [non-inverting] and the mastoid on each side [ipsilateral as inverting; contralateral as ground electrode]. The impedance was below 10 kΩ for all recordings. The data acquisition program rejected traces contaminated by high-amplitude artifacts (voltage greater than ± 23.80 μV). 1300 sweeps were averaged to complete each run, and two consecutive averages were obtained for each ear. The right and left ears were then averaged to obtain a single measurement from each subject. The EEG was amplified and band-pass filtered from 30 to 1500 Hz.

Infants who passed the hearing screening test continued on to the forward-masking protocol, presented by using a pair of 100 μs click stimuli at 80 dB, nHL. The time between the “masker” and “probe” clicks was varied with intervals of 8, 16, and 32 ms and presented in blocks, beginning with the 32 ms condition, followed by the 16 ms and 8 ms conditions. Responses to the second (probe) stimulus were measured (wave I, III, and V latencies). The pairs of click stimuli were delivered to each ear by insert transducers with a presentation rate of 11.7/sec. The recording epoch was 74.67 ms. For each condition, two replications were added, yielding an average waveform for 2600 sweeps per ear. Individual waves for each condition were identified and marked by trained technicians. Values for the right and left ears were averaged so that each infant was assigned a single value for each condition, derived from a total of 5200 sweeps.

Latencies for waves I, III, and V were used to calculate the central-to-peripheral (C-P ratio), which is the ratio between the III-V and I-III interpeak intervals ([latency V- latency III]/[latency III- latency I]). Of the 391 neonates with ABR testing, maternal lead was available for 343 at mid-pregnancy and 362 at late-pregnancy; cord blood lead was available for 321.

### Grating Visual Acuity (VA)

Visual acuity improves throughout in infancy and childhood as the visual pathway becomes myelinated [29]. Grating VA was measured in six-week-old infants using a preferential looking test procedure with Teller Acuity Cards. Luminance of the ambient lighting was kept constant at 85 candelas/m^2^ from overhead diffuse fluorescent lights. VA testing was performed by three well-trained examiners who were blinded to the infants’ lead exposure status. Infants were held upright by their mothers facing a Teller Acuity Test stage placed 38 cm away. Examiners presented infants with a series of mounted gray prints, with black and white vertical gratings to one side and a blank to the other side, through a rectangular opening in the stage. Gratings ranged from coarse to fine (0.44–27 cycles/degree) and cards were of approximately 35 % reflectance. Cards were presented in order of lower to higher spatial frequencies (wider to narrower gratings) and so gratings were located on both the left and right sides of the print for each frequency. Examiners watched the child’s eye movements through a small central aperture in the stage and decided, based on the infant’s looking behavior, which cards could be seen by the infant. If looking responses were unclear, examiners would repeat the presentation several times until a confident judgment could be made about whether the grating could be seen. Acuity was estimated as the spatial frequency of the finest grating that the infant could resolve, as indicated by the child’s consistent looking toward the location of that grating upon repeated presentations of the card. If an examiner had low confidence in his or her acuity estimate, a second examiner, blinded to the results of the first testing, would re-test the infant. If the infant’s condition was determined to be unsuitable for testing that day, parents were asked to return for testing another day. Grating VA testing was performed for 1148 infants. Of these, maternal lead was available for 1038 at mid-pregnancy and 1058 at late-pregnancy, and cord blood lead was available for 949.

### Covariates

Sex, gestational age, birth weight, and head circumference were all recorded at the time of birth. Gestational age was estimated from last menstrual period. Cord blood iron status was defined using serum ferritin, which was measured by chemiluminescent immunoassay (IMMULITE, Diagnostic Products) and categorized into deficient or normal (≤75 and >75 μg/L). Serum ferritin values >370 μg/L were excluded due to possibility of inflammation or infection.

### Statistical analysis

SAS version 9.3 (SAS Institute, Cary, NC) was used for all analyses. Descriptive statistics, frequencies, and correlations for all variables of interest were examined. Generalized Linear Models (GLM) were used to evaluate associations between categories of prenatal blood lead exposure at the three time points and either newborn ABR C-P ratio (for 32, 16, and 8 ms conditions) or six-week grating VA scores. Crude, partially adjusted (for sex, age at testing, cord ferritin), and fully adjusted (additional adjustments for birth weight and head circumference) models were analyzed for all relationships of interest. Sensitivity analyses excluding infants with low serum ferritin were also investigated to test the strength of the relationships between lead exposure and sensory function without the possible confounding influence of iron deficiency. To address the possibility that the few extremely high lead levels observed may have been due to laboratory contamination or blood clotting, additional sensitivity analyses excluding those high lead levels were also completed.

## Results

Distributions of the prenatal lead concentrations can be found in Table 1. Late-pregnancy lead and cord lead concentrations were significantly correlated; Spearman rank ρ = 0.43, *p* < 0.0001 and ρ = 0.30, *p* < 0.0001 for the infants with ABR or VA samples, respectively. Mid- and late-pregnancy lead concentrations were not significantly correlated, nor were mid-pregnancy and cord lead concentrations. Full characteristics of the study sample are shown in Table 2.

**Table 1:** Distribution of prenatal lead concentrations in the study sample at three time points (μg/dL)

	Infants with Newborn ABR Data							Infants with 6-Wk Grating VA Data						
Lead exposure	N	GM (SD)^a^	Percentile					N	GM (SD)^a^	Percentile				
			50th	75th	90th	95th	Max.			50th	75th	90th	95th	Max.
Mid-pregnancy^b^	343	2.4 (2.5)	2.9	4.4	5.5	6.4	9.0	1038	2.4 (2.6)	2.9	4.4	6.3	8.2	19.0
Late-pregnancy^c^	362	2.7 (2.3)	3.0	4.5	6.2	7.2	76.0	1058	2.9 (2.2)	3.3	4.7	6.4	7.7	69.8
Cord^d^	321	<LOQ	<LOQ	3.2	5.0	5.7	24.7	949	<LOQ	2.1	3.3	4.7	5.5	13.5

^a^ *GM* geometric mean, *SD* standard deviation

^b^Mean (SD) gestational age at mid-pregnancy visit is 15.7 (2.2) weeks for ABR subset and 15.5 (1.9) weeks for VA data

^c^Mean (SD) gestational age at late-pregnancy visit is 38.8 (1.3) weeks for ABR subset and 39.3 (1.3) weeks for VA data

^d^Mean (SD) gestational age at birth is 39.2 (1.1) weeks for ABR subset and 39.7 (1.1) weeks for VA data

**Table 2:** Study sample characteristics

	Infants with Newborn ABR Data		Infants with 6-Wk Grating VA Data	
Variable	N	Median (5^th^, 95^th^ percentile)	N	Median (5^th^, 95^th^ percentile)
Gestational age (wks) at mid-pregnancy visit	353	15.0 (13.0, 19.0)	1054	15.0 (13.0, 19.0)
Gestational age (wks) at late-pregnancy visit	352	39.0 (37.0, 41.0)	1054	39.4 (36.9, 41.3)
Gestational age (wks) at birth	353	39.0 (37.0, 41.0)	1058	39.7 (37.9, 41.4)
Birth weight (g)	361	3400 (2850, 4100)	1054	3400 (2750, 4000)
Head circ. (cm)	361	34.1 (31.9, 36.0)	1030	34.1 (32.0, 36.0)
Age at testing (days)	362	2.2 (1, 4)	1058	43 (41, 89)
		N (%)		N (%)
Sex (male)	350	183 (52.3)	1053	555 (52.5)
Low cord ferritin	360	99 (27.5)	1055	298 (28.3)

Crude, semi-adjusted, and adjusted GLM model results are shown in Table 3. Maternal lead in late-pregnancy was significantly associated with both tests of infant sensory function.

**Table 3:** GLM results for associations between prenatal lead and infant ABR C-P ratio^a^ and grating VA

	Crude		Semi-adjusted^b^		Fully-adjusted^c^	
	N	β (95 % CI)^d^	N	β (95 % CI)^d^	N	β (95 % CI)^d^
ABR C-P ratio						
Mid-pregnancy lead	304		292		292	
High (>3.8 μg/dL)		0.02 (−0.00–0.05)^†^		0.02 (−0.01–0.05)		0.02 (−0.01–0.05)
Med. (2–3.8 μg/dL)		0.02 (−0.01–0.05)		0.02 (−0.01–0.05)		0.02 (−0.01–0.05)
Low (<2 μg/dL); Reference		p-trend = 0.09^†^		p-trend = 0.12		p-trend = 0.13
Late-pregnancy lead	315		304		304	
High (>3.8 μg/dL)		0.04 (0.02–0.07)^**^		0.05 (0.02–0.08)^***^		0.05 (0.02–0.07)^**^
Med. (2–3.8 μg/dL)		0.03 (0.00–0.05)^†^		0.03 (0.01–0.06)^*^		0.03 (0.00–0.06)^**^
Low (<2 μg/dL); Reference		p-trend = 0.002^**^		p-trend < 0.001^***^		p-trend = 0.002^**^
Cord lead	277		267		267	
High (>3.2 μg/dL)		0.00 (−0.03–0.03)		0.00 (−0.02–0.03)		0.00 (−0.03–0.03)
Med. (2–3.2 μg/dL)		−0.00 (−0.03–0.02)		−0.00 (−0.03–0.03)		−0.00 (−0.03–0.03)
Low (<2 μg/dL); Reference		p-trend = 0.99		p-trend = 0.81		p-trend = 0.88
Grating VA						
Mid-pregnancy lead	1000		990		961	
High (>3.8 μg/dL)		0.00 (−0.06–0.08)		−0.03 (−0.09–0.03)		−0.02 (−0.08–0.04)
Med. (2–3.8 μg/dL)		0.02 (−0.05–0.09)		0.02 (−0.04–0.08)		0.03 (−0.03–0.09)
Low (<2 μg/dL); Reference		p-trend = 0.90		p-trend = 0.36		p-trend = 0.52
Late-pregnancy lead	1019		1016		985	
High (>3.8 μg/dL)		−0.12 (−0.19--0.05)^**^		−0.08 (−0.14--0.02)^*^		−0.09 (−0.15--0.02)^**^
Med. (2–3.8 μg/dL)		−0.08 (−0.16--0.01)^*^		−0.07 (−0.13--0.01)^*^		−0.07 (−0.13--0.01)^*^
Low (<2 μg/dL); Reference		p-trend = 0.002^**^		p-trend = 0.01^*^		p-trend = 0.009^**^
Cord lead	916		916		888	
High (>3.2 μg/dL)		−0.02 (−0.10–0.05)		−0.03 (−0.09–0.03)		−0.03 (−0.10–0.03)
Med. (2–3.2 μg/dL)		−0.05 (−0.12–0.02)		−0.05 (−0.11–0.01)^†^		−0.05 (−0.11–0.01)^†^
Low (<2 μg/dL); Reference		p-trend = 0.45		p-trend = 0.25		p-trend = 0.22

^a^32 ms masking condition

^b^Adjusted for sex, age at testing, cord blood iron status

^c^Adjusted for sex, age at testing, cord blood iron status, gestational age, birth weight, head circumference

^d^Effect estimates are reported in cycles/degree for VA; ABR C-P ratio has no units (ms/ms)

† *p* < 0.10; * *p* < 0.05; ** *p* < 0.01; *** *p* < 0.001

**Fig. 1:**
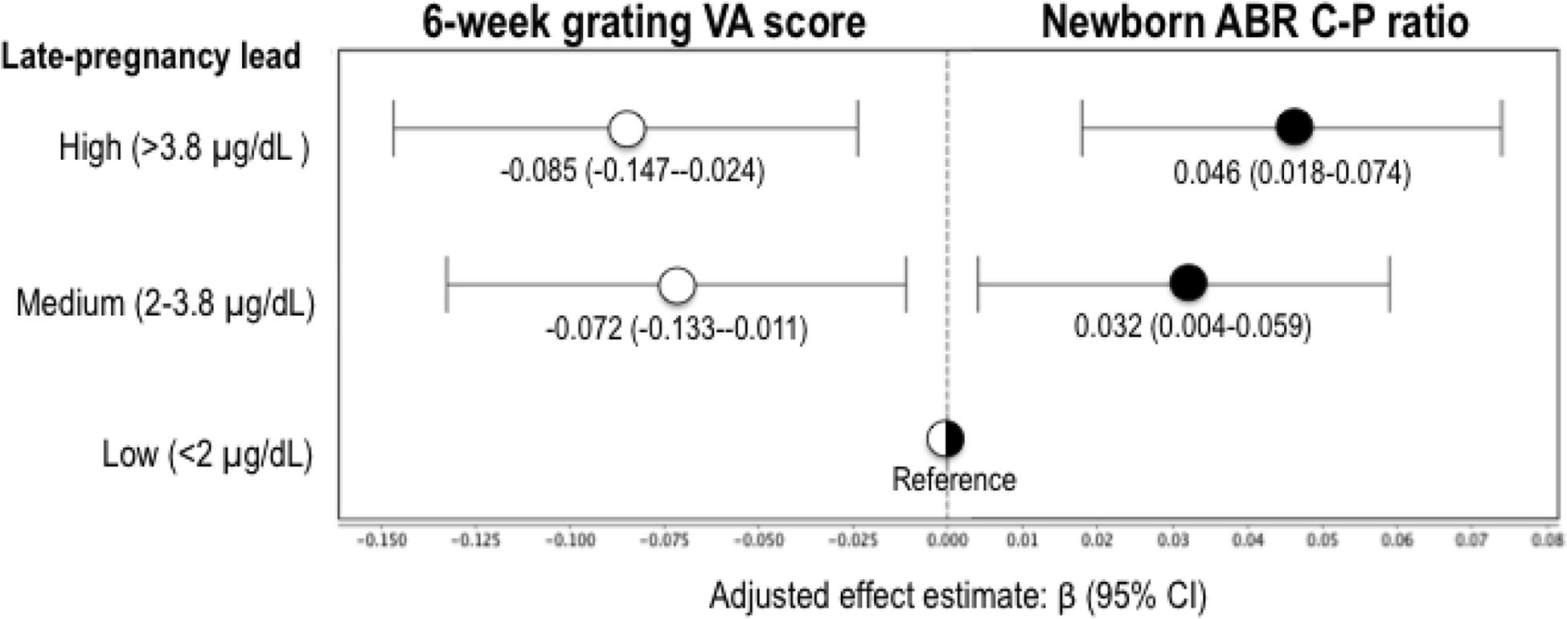
Late-pregnancy maternal lead level and six-week grating VA and newborn ABR C-P ratio^a^. ^a^VA and ABR C-P have different scales; Effect estimates are reported in cycles/degree for VA; ABR C-P ratio has no units (ms/ms)

At the 32 ms masker delay condition, late-pregnancy lead levels were significantly positively associated with newborn ABR C-P ratios, with and without adjustment for covariates. In adjusted analyses, mean C-P ratios were 4.6 % higher (95 % CI: 1.8–7.4 %) and 3.2 % higher (95 % CI: 0–5.9 %), for infants whose mothers had late-pregnancy lead >3.8 μg/dL and lead =2–3.8 μg/dL, respectively, than for infants whose mothers had late-pregnancy lead <2 μg/dL (p-trend =0.002) (Fig. 1). ABR C-P ratios were not associated with mid-pregnancy or cord lead levels. Findings for the 8 ms and 16 ms conditions did not reach statistical significance and are presented in Additional file 1: Table S1. To confirm that the ABR C-P findings were, in fact, due to longer latencies in the inner portions of the auditory pathway, as opposed to shorter latencies in the outer portions of the pathway, we analyzed interpeak intervals I-III and III-V individually with prenatal lead level. The results confirmed that the higher C-P ratios were primarily due to longer wave III-V interpeak intervals (Additional file 1: Table S2).

Grating VA scores were lower with higher late-pregnancy lead level. In adjusted analyses, mean VA scores were 8.5 % lower (95 % CI: 2.4–14.7 %) and 7.2 % lower (95 % CI: 1.1–13.3 %), for infants whose mothers had late-pregnancy lead >3.8 μg/dL and lead =2–3.8 μg/dL, respectively, than for infants whose mothers had late-pregnancy lead <2 μg/dL (p-trend =0.009) (Fig. 1). Similar to the ABR results, no statistically significant associations were observed for VA and levels of mid-pregnancy lead or cord lead.

Sensitivity analyses, with low ferritin infants excluded, confirmed the ABR results. C-P ratios were significantly higher in infants whose mothers had higher blood lead levels late in pregnancy, with similar trends, statistical significance, and directions of association as were seen with the full data set; adjusted β (95 % CI) =0.06 (0.03–0.09), 0.04 (0.01–0.08) for infants whose mothers had late-pregnancy lead >3.8 μg/dL and lead =2–3.8 μg/dL, respectively, compared to infants whose mothers had late-pregnancy lead <2 μg/dL (p-trend < 0.001). The VA results were attenuated with the exclusion of low ferritin infants, though overall trends and directions of associations remained consistent; adjusted β (95 % CI) = −0.07 (−0.14–0.01), −0.05 (−0.13–0.02) for infants whose mothers had late-pregnancy lead >3.8 μg/dL and lead =2–3.8 μg/dL, respectively, compared to infants whose mothers had late-pregnancy lead <2 μg/dL (p-trend = 0.096).

Sensitivity analyses excluding the extremely high lead levels led to the deletion of 8 values (1 from the ABR/late-maternal lead analysis, 1 from the ABR/cord lead analysis, 6 from the VA/late-maternal lead analysis). Exclusion of these values had no notable effect on the results. ABR C-P ratios remained significantly higher in infants whose mothers had higher blood lead levels late in pregnancy, with similar trends, statistical significance, and directions of association as were seen with the full data set; adjusted β (95 % CI) =0.05 (0.02–0.07), 0.03 (0.00–0.06) for infants whose mothers had late-pregnancy lead >3.8 μg/dL and lead =2–3.8 μg/dL, respectively, compared to infants whose mothers had late-pregnancy lead <2 μg/dL (p-trend = 0.002). VA scores also remained significantly higher in infants whose mothers had higher blood lead levels late in pregnancy, with similar trends, statistical significance, and directions of association as were seen with the full data set; adjusted β (95 % CI) = −0.09 (−0.15--0.03), −0.07 (−0.13--0.01) for infants whose mothers had late-pregnancy lead >3.8 μg/dL and lead =2–3.8 μg/dL, respectively, compared to infants whose mothers had late-pregnancy lead <2 μg/dL (p-trend = 0.008).

## Discussion

Despite the relatively low lead levels in our sample, maternal blood lead at or near term was significantly associated with deficits in auditory and visual sensory outcomes in early infancy. Significant associations were not observed for lead levels at time of recruitment (mid-pregnancy) or cord lead levels. Neonates (average two days old) whose mothers had higher lead levels in late pregnancy were more likely to have higher C-P ratios on ABR testing, consistent with impaired myelination of the innermost portions of the auditory pathway. Neonates whose mothers had higher lead levels in late pregnancy were more likely to have lower grating VA scores, consistent with impaired myelination of the visual tract. Sensitivity analyses excluding infants with low iron stores at birth confirmed the robustness of the ABR results but showed some attenuation of the findings for grating VA. Additional sensitivity analyses with extremely high blood levels excluded, in case of laboratory contamination or blood clotting, had no effect on either the ABR or the VA findings.

Given that the late-pregnancy blood sample was collected very near term, it was somewhat unexpected that the cord blood lead results were not more similar to the late-pregnancy lead results. We believe this discrepancy is likely a result of the large number of cord lead samples that were < LOQ (51 % for the ABR sample and 45 % for the VA sample). While it is quite common for lead in cord blood to be lower than maternal blood lead taken in late pregnancy or at birth [9, 30, 31], the relatively low lead levels in our sample coupled with the relatively high LOQ resulted in a large reference group and smaller medium and high lead exposure groups with which to compare it. This may have limited our ability to assess the effect of cord lead level on our outcomes of interest.

Little is known about the effects of prenatal lead exposure on auditory function in humans. We identified three other studies that looked at prenatal lead exposure and auditory-related outcomes in children. Dietrich and colleagues found that higher prenatal blood lead levels, measured at the first prenatal visit, were associated with poorer central auditory processing at five years, as measured by the Filtered Word Subtest (FWS) of the SCAN, a screening test for auditory processing disorders [18]. Two additional studies by Rothenberg et al. found that maternal blood lead at 20 weeks was associated with increased ABR interpeak intervals in one-month-olds (wave I-III) and five-to-seven-year-olds (waves I-III and III-V) [21, 22]. Prenatal lead levels in these studies averaged three to four times higher than in our study (8.2 μg/dL [18] and 8.1 μg/dL [21, 22] versus 2.4 and 2.7 μg/dL).

Other studies have considered associations between blood lead levels in early childhood and various measures of auditory function. Higher concurrent blood lead levels were associated with longer ABR peak latencies in children aged one to six years [13], and longer interpeak intervals at 12 and 48 months [22]. Blood lead levels in 1- to 5-year-olds were significantly positively associated with ABR wave latencies at a five-year follow-up [17]. Additionally, both neonatal blood lead at 10 days and average blood lead concentrations (taken quarterly over the first five years of life) had negative effects on auditory processing in five-year-olds [18]. Thus, higher blood lead levels in young children seem to be consistently associated with auditory deficits.

Blood lead levels in older children may also be associated with deficits in auditory function, though the results are mixed. Higher concurrent blood lead levels were associated with increased interpeak intervals [32], wave I latencies [23], and hearing thresholds [23, 33, 34]. However other studies found no association between concurrent blood lead and ABRs in older children [14–17, 22]. Two studies of multi-age cohorts, ranging from toddlers to teenagers, without age-specific analyses, also found no association between concurrent blood lead and auditory function [19, 20].

Studies in monkeys have yielded similar results. Early life or gestational lead exposure produced long-term changes in ABR latencies, but later exposure had less effect. Rhesus monkeys exposed to very high lead levels (55–122 μg/dL) during gestation and shortly after birth had significantly longer ABR wave latencies, even after cessation of exposure [35, 36]. In contrast, monkeys who were exposed to high levels of lead postnatally, during the first two years of life, did not show reductions in auditory function, as measured by tympanometry, otoacoustic emissions, and ABR [37].

For visual function, there are even fewer studies in humans. To our knowledge this is the first study to examine prenatal lead exposure and grating visual acuity. A few other studies have examined prenatal or childhood lead exposure and other measures of visual function. One study found increased a- and b-wave amplitudes of scotopic electroretinograms of 7- to 10-year-olds with higher first trimester prenatal lead exposure, indicating that lead may target the developing retina [38]. Another study examined concurrent lead exposure and visual function in 6- to 12-year-old children, using pattern reversal visual evoked potentials (VEP), and found no significant effects [17]. However, the same study found that blood lead levels five years prior may be associated with VEP measures [17]. A third study reported significant negative correlations between visual acuity and concurrent blood lead in 6-year-olds [39]. The same study also showed that visual contrast sensitivity was an important confounder in models of blood lead and neurobehavioral tests [39]. Very high blood lead levels in childhood have also been associated with night blindness in children [40, 41].

Rhesus monkeys exposed to high levels of lead (24–37 μg/dL) both pre- and postnatally had lower amplitudes and longer latencies following flash VEP [42], as well as increased b-wave amplitudes for ERG [42, 43]. Monkeys exposed to high postnatal lead exposure showed deficits in temporal visual function [44], while another study found no effect of postnatal lead on photopic spatial acuity development [45].

Therefore, the available human and non-human primate studies suggest that lead can have negative effects on auditory and visual function, especially when exposure occurs prenatally or in early infancy. The brain is rapidly developing during these periods, and environmental insults occurring during them have the potential for long-lasting effects. Our study supports and augments this body of work by providing evidence of deficits in auditory and visual functioning at much lower lead levels than previously investigated.

One mechanism by which lead may contribute to auditory and visual system deficits is through the targeting of oligodendrocytes. Oligodendrocytes are responsible for synthesizing and maintaining myelin in the central nervous system (CNS). Myelin sheaths surround neuronal axons and facilitate transmission of nerve impulses. *In vitro* experiments show that oligodendrocytes may be the most lead-sensitive cell type in the brain [46] and that lead, at high exposure levels, may even lead to de-myelination of neuronal axons [47]. Dose-response studies in rats further report that early oligodendrocyte progenitor cells are more sensitive to lead than mature oligodendrocytes and that exposure to lead during development can delay their differentiation [48]. Thus, lead exposure may impact brain development by interfering with the timing of oligodendrocyte progenitor maturation [48]. Additionally, exposure to a metals mixture (lead, arsenic, and cadmium) in developing rats (*in utero* to two months post-weaning) led to a reduction in myelin thickness and axon-density in the optic nerve and enhanced apoptosis of cells containing myelin-related proteins [49].

Our ABR results support the hypothesis that lead interferes with myelination. The C-P ratio indicates whether nerve conduction and synaptic transmission are different in the central versus distal components of the auditory pathway [27]. Since the process of myelination is centripetal, the C-P ratio is expected to be higher with a disorder of myelination [27]. We observed higher C-P ratios with higher maternal lead levels late in pregnancy but not at mid-pregnancy. Myelination of sensory tracts in the fetal brain does not begin until late in pregnancy [29, 50]. Our results thus make sense in terms of the time course of auditory system myelination in fetal development.

A plausible alternative mechanism for the auditory findings is that increased prenatal lead exposure may lead to decreased auditory brainstem pathway length. Rothenberg and colleagues investigated this by using head circumference as a surrogate measure for pathway length. They found inverse associations between that head circumference and prenatal lead level [22]. To test this alternative hypothesis in our sample, we similarly analyzed the effects of prenatal lead on head circumference. There were no statistically significant differences in head circumference related to lead level. For visual acuity, Fox and colleagues have shown that low-level gestational lead exposure can affect processes in the retina that do not involve myelination or retinal glial cells, such as increased neurogenesis of rod photoreceptors and rod bipolar cells, and decreased retinal dopamine synthesis, utilization, and release [51]. If low-level prenatal lead exposure can induce negative effects on the retina, there is obviously the potential for deficits in visual function. The Teller Acuity Card procedure used here to assess grating VA does not allow us to distinguish whether the observed decreases in VA are a result of lead-induced effects on myelination of the visual pathway, changes in retinal processes, or some other yet unknown mechanism.

This study is limited by a relatively high LOQ for the lead measurements. This restricts our ability to use lead as a continuous variable and to relate variation in lead levels within the 0–2 μg/dL range to our outcomes of interest. The clinical significance of our statistically significant, but relatively subtle, sensory function deficits early in infancy is unclear. Additional follow-up throughout infancy and later in childhood is warranted. Although examiners were highly trained, assessing very young infants increases the chances of error, especially for the visual acuity measurement. Furthermore, solely presenting the TAC cards in descending order (wider to narrower gratings), as recommended in the testing manual, may have resulted in habituation, possibly confounding the acuity estimate. This can be addressed by presenting the cards in both ascending and descending sequence, however limitations in the attention span of 6-week-old infants necessitated that the test be completed quickly, thus this was not an option. Additionally, there may be confounding by iron deficiency [52], which we addressed in two ways: 1) controlling for cord blood iron status in our models and 2) performing sensitivity analyses where infants with low iron stores were excluded.

This analysis also has a number of strengths. This is only the fourth study to examine the effects of prenatal lead exposure on auditory brainstem response in humans and the first to do so regarding grating visual acuity. The lead levels in this study are lower than those that have been examined in the previously published literature, making the findings relevant for current day exposure levels. The tests of sensory function, ABR and grating VA, provide non-invasive ways of measuring auditory and visual function in infants, as well as providing insights into the levels of myelination of those pathways. The collection of blood lead from three time points during pregnancy gives a more accurate portrayal of the timing of fetal lead exposures.

## Conclusions

This work provides evidence of delayed auditory and visual systems maturation in infants with higher prenatal lead exposure during late pregnancy, even at relatively low levels. Both systems start myelinating in late gestation and mature rapidly in infancy. Higher ABR C-P ratios and lower grating VA scores suggest that even low-level lead exposure in late pregnancy has negative effects on sensory system myelination early in life.

Visual and auditory system development in infancy provides the foundation for many subsequent learning processes, such as communication, language, and reading development [11, 12]. Therefore, delays or altered timing of sensory systems myelination with prenatal lead exposure may potentially contribute to poorer developmental outcomes later on.

## Abbreviations

ABR, Auditory brainstem response; BAEP, Brainstem auditory evoked potential; CI, confidence interval; CNS, Central nervous system; C-P ratio, Central-to-peripheral ratio; FWS, Filtered word subtest; LOD, limit of detection; LOQ, limit of quantification; VEP, Visual evoked potentials

## Additional file

Additional file 1: Table S1. GLM results for associations between prenatal lead and infant ABR C-P ratio for the 8 ms and 16 ms masking conditions. **Table S2:** GLM results for associations between prenatal lead and infant ABR interpeak intervals for the 32 ms masking condition. (DOCX 18 kb)
