# Supplementary material for: Low-level prenatal lead exposure and infant sensory function
Source: Environ Health. 2016 Jun 7;15:65. doi: 10.1186/s12940-016-0148-6 (PMC4897806; doi:10.1186/s12940-016-0148-6)
Supplement: Additional file 1: Table S1. — GLM results for associations between prenatal lead and infant ABR C-P ratio for the 8 ms and 16 ms masking conditions. Table S2: GLM results for associations between prenatal lead and infant ABR interpeak intervals for the 32 ms masking condition. (DOCX 18 kb) [file 12940_2016_148_MOESM1_ESM.docx]

**Table S1**

GLM results for associations between prenatal lead and infant ABR C-P ratio for the 8 ms and 16 ms masking conditions

|  | **Crude** | | **Semi-adjusted ^a^** | | **Fully-adjusted ^b^** | |
| --- | --- | --- | --- | --- | --- | --- |
| **Pb exposure** | **N** | **β (95% CI)** | **N** | **β (95% CI)** | **N** | **β (95% CI)** |
| **8 ms masking condition** |  |  |  |  |  |  |
| **Mid-pregnancy** | 267 |  | 257 |  | 256 |  |
| High (>3.8 ug/dL) |  | 0.03 (-0.00-0.07) ^†^ |  | 0.04 (-0.00-0.07) ^†^ |  | 0.04 (-0.00-0.07) ^†^ |
| Med. (2-3.8 ug/dL) |  | 0.01 (-0.02-0.05) |  | 0.01 (-0.02-0.05) |  | 0.01 (-0.02-0.05) |
| Low (<2ug/dL); Reference |  | p-trend= 0.07 ^†^ |  | p-trend= 0.06 ^†^ |  | p-trend= 0.07 ^†^ |
| **Late-pregnancy** | 279 |  | 270 |  | 269 |  |
| High (>3.8 ug/dL) |  | -0.01 (-0.05-0.02) |  | 0.01 (-0.02-0.05) |  | -0.02 (-0.05-0.02) |
| Med. (2-3.8 ug/dL) |  | 0.02 (-0.01-0.06) |  | 0.01 (-0.02-0.05) |  | 0.02 (-0.01-0.06) |
| Low (<2ug/dL); Reference |  | p-trend= 0.36 |  | p-trend= 0.47 |  | p-trend= 0.28 |
| **Cord** | 250 |  | 242 |  | 241 |  |
| High (>3.2 ug/dL) |  | -0.03 (-0.06-0.01) |  | -0.03 (-0.06-0.01) |  | -0.03 (-0.06-0.01) |
| Med. (2-3.2 ug/dL) |  | -0.03 (-0.06-0.01) |  | -0.03 (-0.06-0.01) |  | -0.03 (-0.06-0.01) |
| Low (<2ug/dL); Reference |  | p-trend= 0.11 |  | p-trend= 0.12 |  | p-trend= 0.13 |
| **16 ms masking condition** |  |  |  |  |  |  |
| **Mid-pregnancy** | 295 |  | 283 |  | 282 |  |
| High (>3.8 ug/dL) |  | 0.03 (-0.01-0.06) |  | 0.03 (-0.01-0.06) |  | 0.03 (-0.01-0.06) |
| Med. (2-3.8 ug/dL) |  | 0.02 (-0.01-0.05) |  | 0.02 (-0.01-0.06) |  | 0.03 (-0.01-0.06) |
| Low (<2ug/dL); Reference |  | p-trend= 0.14 |  | p-trend= 0.13 |  | p-trend= 0.13 |
| **Late-pregnancy** | 307 |  | 296 |  | 295 |  |
| High (>3.8 ug/dL) |  | 0.02 (-0.01-0.05) |  | 0.02 (-0.02-0.05) |  | -0.02 (-0.05-0.02) |
| Med. (2-3.8 ug/dL) |  | 0.02 (-0.02-0.05) |  | 0.02 (-0.02-0.05) |  | 0.02 (-0.01-0.06) |
| Low (<2ug/dL); Reference |  | p-trend= 0.29 |  | p-trend= 0.42 |  | p-trend= 0.26 |
| **Cord** | 274 |  | 264 |  | 263 |  |
| High (>3.2 ug/dL) |  | -0.00 (-0.04-0.03) |  | -0.01 (-0.04-0.03) |  | -0.00 (-0.04-0.03) |
| Med. (2-3.2 ug/dL) |  | 0.01 (-0.03-0.05) |  | 0.01 (-0.03-0.05) |  | 0.01 (-0.03-0.05) |
| Low (<2ug/dL); Reference |  | p-trend= 0.90 |  | p-trend= 0.86 |  | p-trend= 0.90 |

a- Adjusted for gender, age at testing, cord blood iron status

b- Adjusted for gender, age at testing, cord blood iron status, gestational age, birth weight, head circumference

† p<0.10; * p<0.05; ** p<0.01; *** p<0.001

**Table S2**

GLM results for associations between prenatal lead and infant ABR interpeak intervals for the 32 ms masking condition

|  | **Crude** | | | **Semi-adjusted ^a^** | | | **Fully-adjusted ^b^** | | |
| --- | --- | --- | --- | --- | --- | --- | --- | --- | --- |
|  |  | **I-III interval** | **III-V interval** |  | **I-III interval** | **III-V interval** |  | **I-III interval** | **III-V interval** |
| **Pb exposure** | **N** | **β (95% CI)** | **β (95% CI)** | **N** | **β (95% CI)** | **β (95% CI)** | **N** | **β (95% CI)** | **β (95% CI)** |
| **Mid-pregnancy** | 304 |  |  | 292 |  |  | 292 |  |  |
| High (>3.8 ug/dL) |  | -0.03 (-0.10-0.05) | 0.06 (0.00-0.12) ^*^ |  | -0.02 (-0.10-0.05) | 0.06 (-0.00-0.12) ^†^ |  | -0.02 (-0.10-0.05) | 0.06 (-0.00-0.11) ^†^ |
| Med. (2-3.8 ug/dL) |  | -0.04 (-0.11-0.03) | 0.04 (-0.01-0.10) |  | -0.04 (-0.11-0.03) | 0.04 (-0.02-0.09) |  | -0.05 (-0.12-0.02) | 0.04 (-0.02-0.09) |
| Low (<2ug/dL); Ref. |  | p-trend= 0.47 | p-trend= 0.04 ^*^ |  | p-trend= 0.51 | p-trend= 0.06 ^†^ |  | p-trend= 0.51 | p-trend= 0.06 ^†^ |
| **Late-pregnancy** | 315 |  |  | 304 |  |  | 304 |  |  |
| High (>3.8 ug/dL) |  | -0.07 (-0.14-0.01) ^†^ | 0.09 (0.04-0.15) ^**^ |  | -0.08 (-0.15--0.00) ^*^ | 0.10 (0.04-0.16) ^***^ |  | -0.07 (-0.14-0.00) ^†^ | 0.10 (0.04-0.16) ^***^ |
| Med. (2-3.8 ug/dL) |  | -0.07 (-0.14-0.01) ^†^ | 0.04 (-0.02-0.09) |  | -0.07 (-0.14-0.01) ^†^ | 0.05 (-0.00-0.11) ^†^ |  | -0.07 (-0.14-0.01) ^†^ | 0.05 (-0.00-0.11) ^†^ |
| Low (<2ug/dL); Ref. |  | p-trend= 0.08 ^†^ | p-trend= 0.001 ^**^ |  | p-trend= 0.05 ^†^ | p-trend <0.001 ^***^ |  | p-trend= 0.07 ^†^ | p-trend <0.001 ^***^ |
| **Cord** | 277 |  |  | 267 |  |  | 267 |  |  |
| High(>3.2 ug/dL) |  | 0.00 (-0.07-0.07) | 0.01 (-0.05-0.07) |  | -0.00 (-0.07-0.07) | 0.02 (-0.04-0.08) |  | 0.00 (-0.07-0.07) | 0.02 (-0.04-0.08) |
| Med. (2-3.2 ug/dL) |  | 0.02 (-0.05-0.09) | 0.00 (-0.06-0.06) |  | 0.01 (-0.06-0.09) | 0.01 (-0.05-0.07) |  | 0.02 (-0.06-0.09) | 0.01 (-0.05-0.07) |
| Low (<2ug/dL); Ref. |  | p-trend= 0.90 | p-trend= 0.71 |  | p-trend=0.99 | p-trend= 0.56 |  | p-trend= 0.89 | p-trend= 0.53 |

a- Adjusted for gender, age at testing, cord blood iron status

b- Adjusted for gender, age at testing, cord blood iron status, gestational age, birth weight, head circumference

† p<0.10; * p<0.05; ** p<0.01; *** p<0.001
